# Ankle-brachial index rather than brachial-ankle pulse wave velocity is associated with cognitive function in older adults undergoing incident hemodialysis: a multicentre cross-sectional study

**DOI:** 10.1080/07853890.2026.2670076

**Published:** 2026-05-18

**Authors:** Kai Zheng, Chuanjun Huang, Yue Wang, Yahui Wu, Jiangling Guo, Cheng Chen, Yizhi Liu, Cheng Lin, Li Qin, Hailong Li, Lizhe Wu, Xinyang Wang, Shixing Meng, Peipei Han, Qi Guo

**Affiliations:** ^a^ Department of Rehabilitation Medicine, Shanghai University of Medicine and Health Sciences Affiliated Zhoupu Hospital, Shanghai, China; ^b^ School of Health Science and Engineering, University of Shanghai for Science and Technology, Shanghai, China; ^c^ Graduate School of Shanghai University of Traditional Chinese Medicine, Shanghai University of Traditional Chinese Medicine, Shanghai, China; ^d^ Department of Rehabilitation Medicine, School of Health, Fujian Medical University, Fuzhou, China; ^e^ Jiading Subdistrict Community Health Center, Shanghai, China

**Keywords:** Arterial stiffness, cognitive function, hemodialysis, chronic kidney disease

## Abstract

**Background and aims:**

Hemodialysis (HD) patients frequently experience cognitive impairment, potentially linked to arterial stiffness. However, the correlation between specific arterial stiffness and cognitive performance in hemodialysis patients has not been confirmed. The aim of this study was to examine the association between cognitive impairment and arterial stiffness in HD patients.

**Methods:**

This cross-sectional study included HD patients undergoing hemodialysis treatment from seven hemodialysis centers in China. We determined arterial stiffness by brachial-ankle pulse wave velocity (baPWV) and ankle-brachial index (ABI) and assessed participants’ cognitive function using the Mini-Mental State Examination (MMSE).

**Results:**

A total of 889 HD participants were enrolled, with a mean age of 61.4 years, 37.6% being female, a median HD duration of 43.7 months, and 16.4% exhibiting cognitive impairment. Compared with the group with normal cognitive function, patients with cognitive impairment had higher baPWV (21.1 ± 5.8 vs 19.3 ± 4.8) and lower ABI (1.06 ± 0.21 vs 1.13 ± 0.17) (*p* < 0.05). In logistic regression analysis, the highest quartile of ABI was found to have a statistically significant association with cognitive impairment compared to the lowest quartile (odds ratio [OR]: 0.576; 95% confidence interval [CI]: 0.335–0.989) (P for trend = 0.031). Additionally, per standard deviation (SD) increase in ABI was associated with lower odds of cognitive impairment (OR: 0.753; 95% CI: 0.620–0.915). However, in baPWV, we did not find a statistically significant difference (OR: 1.155; 95% CI: 0.964–1.383).

**Conclusions:**

Lower ABI, which is indicative of blockage of blood vessels in the lower limbs, is associated with declining cognitive function in patients on hemodialysis.

**Clinical trial registration:**

ChiCTR1900027039.

## Introduction

End Stage Renal Disease (ESRD) represents the terminal phase of chronic kidney disease (CKD), which necessitates the use of renal replacement therapy. Hemodialysis (HD) is the predominant form of renal replacement therapy, undergone by over 80% of patients with end-stage renal disease [1]. Due to the increasing global life expectancy and the prevalence of chronic conditions such as hypertension and diabetes, the incidence of CKD is steadily increasing. Consequently, there is also an increase in the number of patients undergoing HD treatment [2]. Although HD technology currently sustains nearly 3 million end-stage renal disease patients worldwide, the overall health condition of these patients is significantly inferior to that of normal older adults of the same age [3,4]. This includes decreased physical mobility, greater disease burden, and heightened risk of mortality [5,6]. Recent research has consistently shown a high prevalence of cognitive impairment among HD patients, affecting 80% of individuals. This rate is nearly three times higher than for the age-matched general population [7]. However, despite the widespread occurrence and the detrimental effects of cognitive impairment on patient decision-making, quality of life, dialysis security, and survival [8–10], the fundamental mechanisms and risk indicators of cognitive impairment in this population remain unknown. Most previous studies have considered the disease to be primarily related to malnutrition and accumulation of neurotoxic substances, but in recent years, arterial stiffness has also been recognised as an additional risk factor for cognitive impairment in dialysis patients [11].

Arterial stiffness, characterised by a reduction in the volume of arteries in response to pressure changes, is a prevalent and chronic vascular disease [12]. This condition arises from a loss of elasticity and compliance in the arteries, and it has emerged as one of the earliest indicators of structural and functional changes in the vessel wall. Chronic kidney disease significantly contributes to arterial stiffness, especially in individuals undergoing HD [13]. The presence of multiple comorbidities, along with typical factors contributing to arterial stiffness (such as advanced age), increases the susceptibility of dialysis patients to this condition. Therefore, arterial stiffness has become a growing concern in patients undergoing long-term HD [14]. Arterial stiffness has been linked to various adverse health outcomes, including reduced quality of life, increased rates of hospitalisation, and heightened risk of cardiovascular mortality [15,16]. Investigating the association between arterial stiffness and cognitive impairment is crucial, given the high prevalence of both conditions among individuals with HD. The ankle-brachial index and brachial-ankle pulse wave velocity are established techniques for non-invasive evaluation of arterial stiffness in clinical settings [17]. Previous studies have examined the association between ABI, baPWV, and cognitive impairment in dialysis patients. However, the findings of these studies are inconclusive and contradictory. Yi et al. [18] found that among a Chinese population, higher baPWV was independently associated with cognitive impairment in peritoneal dialysis patients, while no such association was observed with ABI. Nevertheless, Nishimura et al. [19] demonstrated that ABI served as a reliable indicator for cognitive impairment in patients undergoing dialysis. Moreover, there is a lack of research investigating the correlation between ABI, PWV, and cognitive impairment in HD patients. Investigating this relationship is crucial to validate whether arterial stiffness can serve as an indicator for screening cognitive impairment in dialysis patients.

Therefore, the primary aim of this study was to examine the association between arterial stiffness and cognitive impairment in Chinese hemodialysis patients using ABI and baPWV measurements. Our hypothesis posited that arterial stiffness (ABI and baPWV) in hemodialysis patients is linked to cognitive impairment.

## Methods

### Study protocol

This study employed a multicenter cross-sectional design, enrolling hemodialysis patients aged ≥18 years from seven dialysis centers in Shanghai, China, between July 2020 and March 2023. Ethical approval was obtained from the Ethics Committee of Shanghai University of Medicine & Health Sciences (approval no. 2019-WJWXM-04-310108196508064467), and the study adhered to the Declaration of Helsinki and its amendments. All participants provided written informed consent and were assessed by trained professionals through in-person questionnaires and objective measurements.

### Patient population

**Figure 1.**
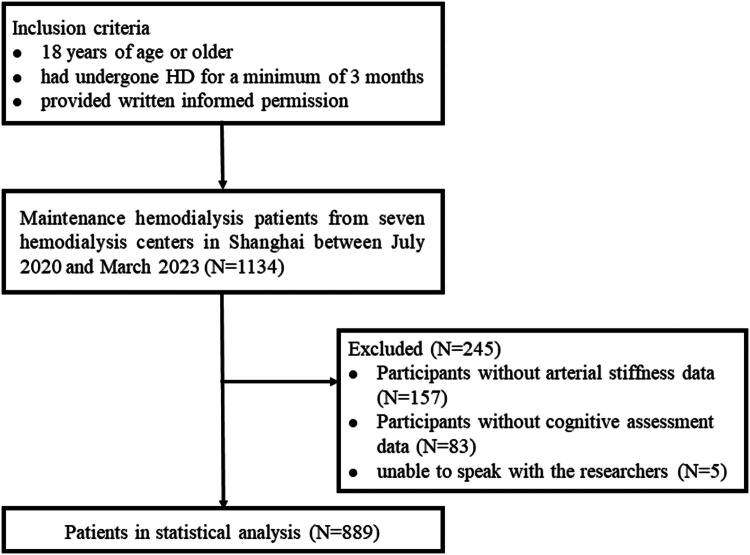
Flow chart of the study.

Eligibility criteria included patients aged ≥18 years who had undergone HD for at least 3 months, with a frequency of 2–3 sessions per week (3–4 h each), and who provided written informed consent. Exclusion criteria were: (1) absence of arterial stiffness data (ABI or baPWV), (2) missing cognitive assessment data, (3) inability to communicate with investigators due to hearing loss or other reasons, or (4) inability to provide informed consent. A flowchart of participant selection is presented in Figure 1.

### Assessment of arterial stiffness relevant indicators

Arterial stiffness indices were assessed using a validated automated device (BP-203PREIII, Omron, Kyoto, Japan), which recorded blood pressure twice after participants had rested in the supine position for at least 5 min. In participants with an arteriovenous fistula, measurements were taken only from the non-fistula arm to prevent confounding of blood pressure values. The ankle–brachial index (ABI) was calculated as the ratio of ankle systolic blood pressure (right and left) to the higher systolic blood pressure of the non-fistula arm. When the right and left ABI values differed, the lower value was used for analysis [20]. For baPWV, pulse waveforms from both brachial and tibial arteries were simultaneously recorded to determine transmission time. BaPWV was defined as the time interval between the initial upstroke of the brachial and tibial waveforms, with transmission distance estimated from body height [21]. The device automatically calculated baPWV as transmission distance divided by transmission time, and the higher side value was used for analysis. To minimise the influence of dialysis on vascular parameters, ABI and baPWV measurements were conducted immediately before the first dialysis session of the week.

### Assessment of cognitive impairment

In this study, cognitive function was assessed using the Chinese version of the Mini-Mental State Examination (MMSE), a widely used tool for evaluating cognitive impairment [22]. This version of the MMSE has been specifically adapted to the linguistic and cultural characteristics of the Chinese population, ensuring greater relevance and accuracy. It is scored on a scale of 0–30, with higher scores indicating better cognitive performance. The MMSE evaluates multiple cognitive domains, including orientation to time (5 points), orientation to place (5 points), registration (3 points), attention and calculation (5 points), recall (3 points), and language (9 points). The cut-off points for assessing cognitive impairment were as follows, based on participants’ educational level: <17 for those who are illiterate, ≤20 for individuals who have completed elementary school, and ≤24 for individuals who have completed middle school or higher [23,24].

### Covariates

All participants underwent a structured face-to-face interview using a standardised questionnaire. Covariates encompassed sociodemographic factors (age, sex, height, weight, marital status, level of education, and duration of hemodialysis), behavioural characteristics (smoking and drinking habits, and daily activity levels), and comorbid chronic conditions. Physical activity was assessed using the short form of the International Physical Activity Questionnaire (IPAQ). Venous blood samples were collected prior to the dialysis session. Further methodological details have been reported previously [5,25–27].

### Statistical analyses

Descriptive statistics were used to summarise the data, expressed as mean ± standard deviation, median (interquartile range [Q1–Q3]), or frequency (percentage) as appropriate. Group comparisons between participants with and without cognitive impairment were performed using Student’s *t* test or Mann–Whitney *U* test for continuous variables, and chi-square test for categorical variables. ABI and baPWV levels were categorised, with the lowest quartile designated as the reference group. Logistic regression was applied to estimate odds ratios (OR) and 95% confidence intervals (CI) and to evaluate the association between arterial stiffness and cognitive impairment. Three regression models were constructed: (a) unadjusted, (b) adjusted for age and sex, and (c) further adjusted for education (<12 years), smoking habits, drinking habits, duration of dialysis, hypertension, diabetes, hyperlipidemia, and IPAQ. P for trend was derived by modelling the median value of each quartile as a continuous variable.

Sensitivity analyses were conducted to assess the robustness of the findings. Given the role of physical activity in both atherosclerosis and cognition [20,28], we examined whether the association persisted after excluding participants with low physical activity to reduce potential reverse causality. To minimise the influence of short dialysis vintage, the association between arterial stiffness and cognitive impairment was reanalysed after excluding participants with <2 years of dialysis. To avoid bias from education-specific thresholds, a uniform MMSE cut-off of <24 was applied regardless of educational level. To account for potential hemodynamic differences related to vascular access type, a sensitivity analysis stratified by access route was conducted. All analyses were conducted using SPSS version 25.0 (IBM Corporation, Armonk, NY, USA), with *p* < 0.05 regarded as statistically significant.

## Results

### Characteristics of the participants

Table 1 presents the characteristics of the sample. Data from 889 participants (334 female; average age: 61.4 ± 12.5 years). The overall prevalence of cognitive impairment, as determined by MMSE scores, was 16.4%. The MMSE scores for the cognitively impaired and cognitively normal groups were 20.0 and 27.4, respectively. In the overall population, individuals with cognitive impairment tended to be older, female, had lower levels of education, pre-dialysis serum creatinine, smoking rates, daily activity levels, and were more likely to have diabetes mellitus, coronary artery disease, and strokes compared to those without cognitive impairment. In addition, individuals with cognitive impairment exhibited lower ABI (1.06 ± 0.21 vs 1.13 ± 0.17) and higher baPWV (21.1 ± 5.8 vs 19.3 ± 4.8) indices in terms of arterial stiffness (*p* < 0.05).

**Table 1.** Baseline characteristics of study participants according to the presence of a cognitive condition.

Variables	Total (*n* = 889)	Cognitive impairment (*n* = 146)	Normal cognitive function (*n* = 743)	*p* values
**Demographics**				
Age (years)	61.4 ± 12.5	67.3 ± 9.6	60.3 ± 12.7	**<0.001**
Female (*n*, %)	334 (37.6%)	70 (47.9%)	264 (35.5%)	**0.005**
Accepted below 12 years of education (*n*, %)	739 (83.1%)	134 (91.7%)	605 (81.4%)	**0.002**
Living alone (*n*, %)	45 (5.1%)	8 (5.5%)	37 (5.0%)	0.801
BMI (kg/m^2^)	23.4 ± 3.8	23.1 ± 3.5	23.4 ± 3.9	0.413
Duration of hemodialysis (months)	43.7 (19.4–95.1)	45.0 (24.5–78.8)	43.5 (18.0–100.4)	0.604
Cause of ESRD (%)				**0.041**
Diabetic	190 (21.4%)	43 (29.9%)	147 (19.8%)	
Hypertensive	138 (15.6%)	26 (18.1%)	112 (15.1%)	
Glomerulonephritis	252 (28.4%)	35 (24.3%)	217 (29.2%)	
Polycystic kidney	53 (6.0%)	8 (5.6%)	45 (6.1%)	
Other	254 (28.6%)	32 (22.2%)	222 (29.9%)	
Types of vascular access				0.096
AVF	784 (88.4%)	120 (83.3%)	664 (89.4%)	
CVC	103 (11.6%)	24 (16.7%)	79 (10.6%)	
Systolic blood pressure (mmHg)	154.1 ± 26.8	157.6 ± 28.4	153.4 ± 26.5	0.088
Diastolic blood pressure (mmHg)	82.6 ± 16.0	81.1 ± 15.7	82.9 ± 16.1	0.205
**Lifestyle variables**				
Smoking (*n*, %)	198 (22.3%)	18 (12.3%)	180 (24.2%)	**0.002**
Drinking (*n*, %)	96 (10.8%)	12 (8.2%)	84 (11.3%)	0.272
IPAQ (Met-min/wk)	1356 (462–3066)	693 (0–2373)	1386 (495–3066)	**0.008**
**Laboratory parameters**				
Hemoglobin (g/L)	109.7 ± 16.9	108.1 ± 17.0	110.0 ± 16.9	0.219
Serum albumin (g/L)	39.2 ± 3.8	38.7 ± 3.8	39.3 ± 3.8	0.105
C-reactive protein(mg/L)	2.9 (1.4–6.0)	2.9 (1.3–6.1)	2.9 (1.5–6.0)	0.881
Serum calcium (g/L)	2.3 ± 0.3	2.2 ± 0.2	2.3 ± 0.3	0.124
Serum phosphorus (mmol/L)	1.9 (1.5–2.4)	1.8 (1.5–2.3)	1.9 (1.5–2.4)	0.418
PTH (pg/mL)	266.1 (142.3–467.9)	272.6 (110.3–460.4)	264.2 (148.6–469.2)	0.537
Total cholesterol (mmol/L)	3.8 (3.2–4.4)	3.7 (3.1–4.3)	3.8 (3.2–4.5)	0.243
Triglycerides (mmol/L)	1.7 (1.2–2.8)	1.6 (1.2–2.9)	1.7 (1.2–2.8)	0.951
High-density lipoprotein (mmol/L)	0.9 (0.8–1.1)	0.9 (0.7–1.2)	0.9 (0.8–1.1)	0.346
Low-density lipoprotein (mmol/L)	2.2 (1.8–2.8)	2.2 (1.8–2.6)	2.3 (1.8–2.8)	0.323
Blood urea nitrogen (mmol/L)	26.0 (21.8–30.2)	25.7 (20.8–29.5)	26.2 (21.9–30.2)	0.379
Serum creatinine (mmol/L)	968.6 (810.5–1149.4)	924.0 (737.0–1122.5)	980.0 (819.0–1154.7)	**0.020**
Kt/V	1.3 (1.2–1.5)	1.3 (1.2–1.5)	1.3 (1.2–1.5)	0.058
**Clinical characteristics**				
Hypertension (*n*, %)	825 (92.8%)	136 (93.2%)	689 (92.7%)	0.858
Diabetes mellitus (*n*, %)	481 (54.1%)	96 (65.8%)	385 (51.8%)	**0.002**
Hyperlipidemia (*n*, %)	256 (28.8%)	39 (26.7%)	217 (29.2%)	0.543
Coronary artery disease (*n*, %)	231 (26.1%)	48 (33.6%)	183 (24.6%)	**0.025**
Stroke (*n*, %)	119 (13.3%)	33 (22.6%)	86 (11.4%)	**<0.001**
**Artery stiffness index**				
Brachial-ankle pulse wave velocity (m/s)	19.6 ± 5.0	21.1 ± 5.8	19.3 ± 4.8	**<0.001**
Ankle brachial index	1.12 ± 0.18	1.06 ± 0.21	1.13 ± 0.17	**<0.001**
Score of mini-mental state examination (points)	26.2 ± 3.9	20.0 ± 4.4	27.4 ± 2.2	**<0.001**

BMI, body mass index; AVF, Arteriovenous Fistula; CVC, Central Venous Catheter; IPAQ, International Physical Activity Questionnaire; Met-min/wk, metabolic equivalent task minutes per week; PTH, parathyroid hormone; Kt/V, fractional clearance index for urea.Bold values indicate statistically significant differences between participants with and without cognitive impairment (*p* < 0.05).

### Association of ABI, baPWV with cognitive function

Table 2 displays the results of the binary logistic regression analysis, elucidating the association between ABI, baPWV, and the occurrence of cognitive impairment. After adjusting for potential confounding factors, including age, sex, education level (<12 years), smoking and drinking habits, duration of dialysis, hypertension, diabetes, hyperlipidemia, and IPAQ. The analysis demonstrated a significant association between ABI (OR 0.753 per 1 SD, 95% CI [0.620–0.915]) and cognitive impairment. Upon stratifying ABI and baPWV into quartiles and incorporating them as categorical variables in the logistic regression models, the analysis indicated that, after adjusting for all pertinent factors, individuals in the highest quartile of ABI had a reduced risk of cognitive impairment compared to those in the lowest quartile. The odds ratios were 0.576 (95% CI: 0.335–0.989). However, there was no significant difference in the association between baPWV and cognitive impairment, whether baPWV was incorporated into the model as a continuous variable (OR 1.155 per 1 SD, 95% CI [0.964–1.383]) or a categorical variable (OR 1.632 , 95% CI [0.903–2.948]). Additionally, the median value of each ABI and baPWV category was utilised as a continuous variable in the multivariate regression model to assess linear trends. A significant trend was observed between ABI and cognitive impairment (*P* for trend = 0.031).

**Table 2.** Association between ABI, baPWV and cognitive impairment.

	Cognitive impairment vs. No cognitive impairment					
	Unadjusted OR (95% CI)	*p* Value	Age- and Sex- adjusted OR (95% CI)	*p* Value	Multivariate-adjusted OR* (95% CI)	*p* Value
(a)						
ABI (per 1SD)	0.653 (0.546–0.781)	**<0.001**	0.743 (0.616–0.897)	**0.002**	0.753 (0.620–0.915)	**0.004**
ABI						
Q1	Ref		Ref		Ref	
Q2	0.710 (0.448–1.126)	0.145	0.812 (0.503–1.309)	0.392	0.869 (0.534–1.416)	0.574
Q3	0.444 (0.267–0.737)	**0.002**	0.606 (0.357–1.029)	0.063	0.640 (0.373–1.099)	0.105
Q4	0.416 (0.249–0.694)	**0.001**	0.549 (0.323–0.933)	**0.027**	0.576 (0.335–0.989)	**0.045**
P for trend		**<0.001**		**0.015**		**0.031**
(b)						
baPWV (per 1SD)	1.368 (1.155–1.597)	**<0.001**	1.193 (1.000–1.424)	**0.050**	1.155 (0.964–1.383)	0.118
baPWV						
Q1	Ref		Ref		Ref	
Q2	1.764 (1.000–3.112)	**0.050**	1.346 (0.749–2.422)	0.321	1.370 (0.753–2.492)	0.302
Q3	1.668 (0.943–2.950)	0.078	1.126 (0.620–2.046)	0.697	1.089 (0.590–2.008)	0.786
Q4	2.833 (1.653–4.854)	**<0.001**	1.750 (0.982–3.118)	0.058	1.632 (0.903–2.948)	0.105
P for trend		**<0.001**		0.074		0.148

Notes: ABI: ankle brachial index; baPWV: brachial ankle pulse wave velocity; OR: odd ratio; CI: confidence interval; 1 SD of ABI = 0.19; 1 SD of ABI = 5.1 m/s.

^*^ Adjust for age, sex, education level (<12 years), smoking habits, drinking habits, duration of dialysis, hypertension, diabetes, hyperlipidaemia, IPAQ.Bold values indicate statistically significant associations between arterial stiffness indices and cognitive impairment (*p* < 0.05).

### Sensitivity analysis

We performed a sensitivity analysis by eliminating patients with poor IPAQ scores in order to investigate the relationship between ABI, baPWV, and cognitive impairment. The findings were consistent with those observed in the entire study population. In addition, the odds ratio for cognitive impairment based on ABI showed a slight decrease from 0.753 (95% CI [0.620–0.915]) to 0.622 (95% CI [0.472–0.820]). However, a similar finding was not observed for baPWV (Supplemental Table S1).

Furthermore, recognising that participants with <2 years of dialysis may not be representative of the broader population, we analysed the data after excluding this subset of the sample. ABI remained significantly associated with cognitive impairment (OR 0.745; 95% CI, 0.590–0.942), whereas no significant association was found with baPWV (OR 1.224; 95% CI, 0.983–1.525). The results remained consistent, with no significant changes observed (Supplemental Table S2).

To further evaluate the robustness of our findings, we conducted a sensitivity analysis using a uniform criterion of MMSE <24 to define cognitive impairment, irrespective of educational levels. The results were largely consistent with our primary analyses, except that the ABI remained significantly associated with cognitive impairment (OR 0.712; 95% CI, 0.582–0.871), while baPWV did not show a significant association (OR 1.108; 95% CI, 0.917–1.338). These findings underscore the robust association between ABI and cognitive impairment, even when using a different diagnostic criterion for cognitive impairment (Supplemental Table S3).

To examine the influence of vascular access type, we stratified the cohort. In the AVF subgroup (*n* = 784), ABI remained independently associated with cognitive impairment (adjusted OR 0.745; 95% CI, 0.609–0.911; *p* = 0.004), but no significant association was observed in the CVC subgroup (Supplemental Table S4).

## Discussion

Our study demonstrated a significant association between lower ABI, a measure of lower extremity vascular blockage, and the presence of global cognitive impairment. This observation aligns with the possibility that arterial stiffness is concurrently linked with poorer cognitive executive and overall function among incident hemodialysis patients.

In this study, the prevalence of cognitive impairment in HD patients was 16.4% as detected by MMSE. A comprehensive meta-analysis of 37 studies (129,849 HD patients) reported the occurrence among HD patients varied between 13% and 81.1%; pooled prevalence was 49.1% (57.3% *via* MoCA, 35.0% *via* MMSE) [29]. The lower prevalence in our study is attributable to distinct criteria [30]. Specifically, we used literacy-based MMSE cut-offs (<17 for illiterate, ≤20 for elementary education, ≤24 for middle school or higher), whereas other MMSE-based studies adopted a uniform cut-off of <24 [31,32]. Furthermore, our larger proportion of younger participants may have contributed to the lower prevalence.

Our study found that after adjustment for age, sex, education, lifestyle and medical variables, cognitive impairment was less prevalent across higher ABI quartiles (OR 0.753 per 1 SD, 95% CI [0.620–0.915]), indicating that ABI is statistically associated with cognitive status. These results are consistent with previous research studies. A previous Japanese study found a significant correlation between reduced ABI and cognitive impairment, as assessed by the MoCA, in patients undergoing hemodialysis [19]. Similarly, a cross-sectional study of 136 Asian HD patients (mean age 59.3 ± 10.5 years, 55.9% male) in Kaohsiung, China, reported lower ABI independently associated with cognitive impairment [33]. The association between arterial stiffness and cognitive impairment may result from compromised integrity of white and gray matter, along with microvascular damage [34,35]. Decreased ABI often signifies vascular endothelial damage and lipid accumulation in the vessel wall, indicating a reduction in overall blood flow, resulting in local tissue ischaemia and hypoxia. These conditions initiate a cascade of adverse effects, including oxidative stress and the buildup of toxic Aβ proteins [36,37], with the brain being particularly vulnerable due to its low resistance and high flow characteristics. Meanwhile, hemodialysis procedure significantly reduces blood supply to the brain, potentially exacerbating brain hypoxia symptoms and impairing cognitive function [38]. Notably, low ABI often causes lower limb claudication, reducing daily activity and increasing sarcopenia risk. Our prior work linked sarcopenia, mobility, and cognition, suggesting cardiovascular factors (e.g. heart rate variability, vascular changes) interact with muscle function and cognition [26,28,39], with ABI potentially influencing cognitive function indirectly through its effects on mobility.

Clinically, carotid-femoral pulse wave velocity (cfPWV) is regarded as the gold standard for assessing aortic stiffness, providing insights into the extent of damage to central blood vessels. BaPWV and cfPWV have similar theoretical foundations, but baPWV is more clinically convenient due to its simplified operation [40]. Our study found no association between baPWV (continuous or categorical) and cognitive impairment in HD patients. Furthermore, even after excluding participants with lower daily activity or <2 years on dialysis, there was still no statistically significant difference between baPWV and cognitive impairment, consistent with the main model. This finding contradicts those of previous studies [18,41]. One study of 72 HD patients found PWV correlated with cognitive impairment using the 3MS assessment [42], but this study comprised hemodialysis patients already under treatment, averaging a duration of 10 years, while our participants had a median dialysis duration of 3.5 years. The accumulation of central blood vessel damage typically transpires over an extended duration [43], resulting in a less apparent association between arterial stiffness and cognitive impairment in the early stages of dialysis. Additionally, it is noteworthy that our research sample primarily comprised younger individuals. Since the influence of ageing and changes in central pressure measurement is more evident in individuals under the age of 50, in contrast to the heightened pulse wave velocity (PWV) observed in older individuals (over 50 years) with elevated levels of aortic stiffness [44]. Moreover, baPWV measurements may be influenced by blood pressure at the time of assessment and may not be accurately determined when ABI values are low [45]. Specifically, in patients with diseases such as atherosclerotic occlusive disease, lower limb arterial stenosis, aortic valve disease, and upper limb arterial stenosis on the side contralateral to the hemodialysis access, the presence of abnormal ABI values can significantly affect baPWV measurements, leading to a marked reduction in baPWV and a “pseudo normalization” phenomenon [46], which could also account for the lack of association between baPWV and cognitive function observed in our study. These factors might have contributed to the lack of correlation between baPWV and cognitive decline observed in our study.

Sensitivity analysis revealed a stronger association between ABI and cognitive impairment among patients with moderate to high daily physical activity levels. This suggests that the correlation between ABI and cognitive impairment in these physically vulnerable patients might be obscured by other factors (such as multiple comorbidities). Likewise, ABI exhibited a stronger association with cognitive impairment in patients with longer durations of dialysis. Although we mitigated acute hemodynamic interference from the arteriovenous fistula by restricting blood-pressure acquisition to the non-fistula arm and selecting the higher bilateral baPWV, the AVF chronically remodels systemic hemodynamics. Sustained elevations in cardiac output and reductions in systemic vascular resistance can diminish apparent arterial stiffness, systematically underestimating ABI and baPWV. Patients with high activity levels, prolonged dialysis duration, and low ABI, therefore, warrant intensified cognitive surveillance. Prospective comparisons of AVF, central venous catheter, and peritoneal dialysis cohorts, together with cuff-based tonometry at sites remote from the vascular access, are needed to quantify modality-specific bias and standardise measurement protocols in renal replacement therapy.

The strengths of our study are as follows: Firstly, it was one of the few large-scale multicentre study (*n* = 889) examining the correlation between arterial stiffness and cognitive impairment in hemodialysis patients. Moreover, recognising that both arterial stiffness and cognitive function are influenced by dialysis treatment, we employed a standardised method to collect the aforementioned indicators in the hours preceding dialysis sessions. Additionally, we addressed the majority of recognised confounding factors in our regression models to assess the independent association between arterial stiffness and cognitive function in this study.

Despite these strengths, there are still some limitations. Firstly, due to the cross-sectional design of this investigation, establishing a causal relationship between arterial stiffness and cognitive impairment may not be feasible. Secondly, we did not assess participants for neuroimaging and genetic factors, such as APOE4, potentially resulting in an underestimation of the occurrence of cognitive impairment. Lastly, our study was confined to Chinese patients undergoing hemodialysis, thus limiting the generalisability of our findings to the broader global hemodialysis community.

## Conclusion

In our study, global cognitive impairment was found to be prevalent among relatively younger incident hemodialysis patients. The association between lower ABI and poorer global cognitive function is consistent with shared pathophysiological pathways (e.g. uremic-atherosclerotic milieu) that may underlie both vascular changes and cognitive decline. Future longitudinal data from large cohorts are required to further explore the causal relationship and potential mechanisms between them to examine cerebrovascular changes associated with arterial stiffness.

## Supplementary Material

Supplemental Material

## Data Availability

The datasets used and/or analysed during the current study are available from the corresponding author on reasonable request.
