# Supplementary material for: Ankle-brachial index rather than brachial-ankle pulse wave velocity is associated with cognitive function in older adults undergoing incident hemodialysis: a multicentre cross-sectional study
Source: Ann Med. 2026 May 18;58(1):2670076. doi: 10.1080/07853890.2026.2670076 (PMC13188536; doi:10.1080/07853890.2026.2670076)
Supplement: Supplemental Material [file IANN_A_2670076_SM8577.docx]

**Supplementary materials**

**Supplementary Table 1**

Association between ABI, baPWV and cognitive impairment (excluding individuals with low daily activity)

**Supplementary Table 2**Association between ABI, baPWV and cognitive impairment (excluding individuals on dialysis <2 years)

**Supplementary Table 3**Association between ABI, baPWV and cognitive impairment (MMSE <24 uniform criterion)

**Supplementary Table 4**Association between ABI, baPWV and cognitive impairment in patients with arteriovenous fistula

TableS1. Association between ABI, baPWV and cognitive impairment (excluding individuals with low levels of daily activity)

|  | Cognitive impairment vs. No cognitive impairment | | | | | |
| --- | --- | --- | --- | --- | --- | --- |
|  | Unadjusted OR (95% CI) | p Value | Age- and Sex- adjusted OR (95% CI) | p Value | Multivariate-adjusted OR* (95% CI) | p Value |
| (a) |  |  |  |  |  |  |
| ABI (per 1SD) | 0.602 (0.469-0.772) | **<0.001** | 0.664 (0.512-0.860) | **0.002** | 0.622 (0.472-0.820) | **0.001** |
| ABI |  |  |  |  |  |  |
| Q1 | Ref |  | Ref |  | Ref |  |
| Q2 | 0.674 (0.374-1.216) | 0.190 | 0.706 (0.385-1.294) | 0.260 | 0.688 (0.370-1.279) | 0.237 |
| Q3 | 0.464 (0.246-0.878) | **0.018** | 0.554 (0.288-1.068) | 0.078 | 0.521 (0.266-1.020) | 0.057 |
| Q4 | 0.363 (0.186-0.708) | **0.003** | 0.435 (0.220-0.63) | **0.017** | 0.399 (0.199-0.803) | **0.010** |
| (b) |  |  |  |  |  |  |
| baPWV (per 1SD) | 1.462 (1.161-1.842) | **0.001** | 1.293 (1.010-1.655) | **0.042** | 1.281 (0.987-1.662) | 0.063 |
| baPWV |  |  |  |  |  |  |
| Q1 | Ref |  | Ref |  | Ref |  |
| Q2 | 1.176 (0.592-2.338) | 0.643 | 0.932 (0.459-1.890) | 0.845 | 0.908 (0.433-1.827) | 0.793 |
| Q3 | 1.328 (0.672-2.626) | 0.414 | 0.937 (0.461-1.907) | 0.958 | 0.897 (0.497-2.058) | 0.771 |
| Q4 | 2.499 (1.308-4.773) | **0.006** | 1.613 (0.809-3.215) | 0.175 | 1.468 (0.798-3.211) | 0.291 |

Notes: ABI: ankle brachial index; baPWV: brachial ankle pulse wave velocity; OR: odd ratio; CI: confidence interval; 1 SD of ABI = 0.19; 1 SD of ABI = 5.1m/s;

* adjust for age, sex, education level (<12 years), smoking habits, drinking habits, duration of dialysis, hypertension, diabetes, hyperlipidemia.

TableS2. Association between ABI, baPWV and cognitive impairment (excluding individuals on dialysis for <2 years)

|  | Cognitive impairment vs. No cognitive impairment | | | | | |
| --- | --- | --- | --- | --- | --- | --- |
|  | Unadjusted OR (95% CI) | p Value | Age- and Sex- adjusted OR (95% CI) | p Value | Multivariate-adjusted OR* (95% CI) | p Value |
| (a) |  |  |  |  |  |  |
| ABI (per 1SD) | 0.647 (0.524-0.800) | **<0.001** | 0.726 (0.583-0.906) | **0.004** | 0.745 (0.590-0.942) | **0.014** |
| ABI |  |  |  |  |  |  |
| Q1 | Ref |  | Ref |  | Ref |  |
| Q2 | 0.524 (0.300-0.916) | **0.023** | 0.624 (0.349-1.116) | 0.112 | 0.721 (0.396-1.313) | 0.285 |
| Q3 | 0.475 (0.266-0.848) | **0.012** | 0.635 (0.347-1.162) | 0.141 | 0.727 (0.386-1.371) | 0.325 |
| Q4 | 0.388 (0.214-0.703) | **0.002** | 0.503 (0.272-0.931) | **0.029** | 0.549 (0.292-1.034) | 0.063 |
| (b) |  |  |  |  |  |  |
| baPWV (per 1SD) | 1.449 (1.190-1.764) | **<0.001** | 1.273 (1.030-1.573) | **0.025** | 1.224 (0.983-1.525) | 0.071 |
| baPWV |  |  |  |  |  |  |
| Q1 | Ref |  | Ref |  | Ref |  |
| Q2 | 1.559 (0.797-3.053) | 0.195 | 1.213 (0.604-2.435) | 0.587 | 1.255 (0.614-2.563) | 0.534 |
| Q3 | 1.746 (0.894-3.410) | 0.103 | 1.201 (0.595-2.423) | 0.610 | 1.152 (0.557-2.383) | 0.703 |
| Q4 | 2.755 (1.459-5.201) | **0.002** | 1.686 (0.847-3.357) | 0.137 | 1.512 (0.740-3.090) | 0.257 |

Notes: ABI: ankle brachial index; baPWV: brachial ankle pulse wave velocity; OR: odd ratio; CI: confidence interval; 1 SD of ABI = 0.18; 1 SD of ABI = 5.0m/s;

* adjust for age, sex, education level (<12 years), smoking habits, drinking habits, hypertension, diabetes, hyperlipidemia, IPAQ.

TableS3. Association between ABI, baPWV and cognitive impairment (according to MMSE <24 for cognitive impairment)

|  | Cognitive impairment vs. No cognitive impairment | | | | | |
| --- | --- | --- | --- | --- | --- | --- |
|  | Unadjusted OR (95% CI) | p Value | Age- and Sex- adjusted OR (95% CI) | p Value | Multivariate-adjusted OR* (95% CI) | p Value |
| (a) |  |  |  |  |  |  |
| ABI (per 1SD) | 0.617 (0.517-0.736) | **<0.001** | 0.730 (0.603-0.884) | **0.001** | 0.712 (0.582-0.871) | **<0.001** |
| ABI |  |  |  |  |  |  |
| Q1 | Ref |  | Ref |  | Ref |  |
| Q2 | 0.534 (0.338-0.841) | **0.007** | 0.615 (0.377-1.002) | 0.051 | 0.635 (0.383-1.052) | 0.078 |
| Q3 | 0.401 (0.244-0.660) | **<0.001** | 0.610 (0.357-1.041) | 0.070 | 0.626 (0.365-1.072) | 0.088 |
| Q4 | 0.367 (0.221-0.609) | **<0.001** | 0.548 (0.320-0.940) | **0.029** | 0.551 (0.318-0.957) | **0.034** |
| (b) |  |  |  |  |  |  |
| baPWV (per 1SD) | 1.359 (1.158-1.595) | **<0.001** | 1.143 (0.954-1.370) | 0.146 | 1.108 (0.917-1.338) | 0.288 |
| baPWV |  |  |  |  |  |  |
| Q1 | Ref |  | Ref |  | Ref |  |
| Q2 | 1.730 (0.970-3.088) | 0.063 | 1.181 (0.640-2.181) | 0.594 | 1.165 (0.625-2.175) | 0.632 |
| Q3 | 2.311 (1.321-4.042) | **0.003** | 1.389 (0.763-2.529) | 0.282 | 1.311 (0.711-2.418) | 0.386 |
| Q4 | 3.148 (1.826-5.426) | **<0.001** | 1.669 (0.920-3.028) | 0.092 | 1.459 (0.794-2.682) | 0.223 |

Notes: ABI: ankle brachial index; baPWV: brachial ankle pulse wave velocity; OR: odd ratio; CI: confidence interval; 1 SD of ABI = 0.19; 1 SD of ABI = 5.1m/s;

* adjust for age, sex, education level (<12 years), smoking habits, drinking habits, hypertension, diabetes, hyperlipidemia, IPAQ.

TableS4. Association between ABI, baPWV and cognitive impairment in hemodialysis patients with arteriovenous fistula (AVF)

|  | Cognitive impairment vs. No cognitive impairment | | | | | |
| --- | --- | --- | --- | --- | --- | --- |
|  | Unadjusted OR (95% CI) | p Value | Age- and Sex- adjusted OR (95% CI) | p Value | Multivariate-adjusted OR* (95% CI) | p Value |
| (a) |  |  |  |  |  |  |
| ABI (per 1SD) | 0.648 (0.538-0.780) | **<0.001** | 0.722 (0.595-0.876) | **0.001** | 0.745 (0.609-0.911) | **0.004** |
| ABI |  |  |  |  |  |  |
| Q1 | Ref |  | Ref |  | Ref |  |
| Q2 | 0.566 (0.340-0.944) | **0.029** | 0.655 (0.385-1.115) | 0.119 | 0.709 (0.411-1.224) | 0.217 |
| Q3 | 0.540 (0.315-0.925) | **0.025** | 0.761 (0.432-1.340) | 0.343 | 0.826 (0.462-1.477) | 0.519 |
| Q4 | 0.409 (0.231-0.724) | **0.002** | 0.535 (0.296-0.967) | **0.038** | 0.601 (0.326-1.106) | 0.102 |
| (b) |  |  |  |  |  |  |
| baPWV (per 1SD) | 1.394 (1.160-1.676) | **<0.001** | 1.239 (1.014-1.513) | **0.036** | 1.207 (0.985-1.479) | 0.070 |
| baPWV |  |  |  |  |  |  |
| Q1 | Ref |  | Ref |  | Ref |  |
| Q2 | 1.398 (0.755-2.586) | 0.286 | 1.122 (0.594-2.119) | 0.723 | 1.179 (0.611-2.275) | 0.624 |
| Q3 | 1.235 (0.658-2.318) | 0.511 | 0.805 (0.414-1.564) | 0.522 | 0.809 (0.408-1.603) | 0.543 |

| Q4 | 2.873 (1.632-5.059) | **<0.001** | 1.858 (1.011-3.413) | **0.046** | 1.795 (0.957-3.368) | 0.068 |
| --- | --- | --- | --- | --- | --- | --- |

Notes: ABI: ankle brachial index; baPWV: brachial ankle pulse wave velocity; OR: odd ratio; CI: confidence interval; 1 SD of ABI = 0.17; 1 SD of ABI = 4.8m/s;

* adjust for age, sex, education level (<12 years), smoking habits, drinking habits, hypertension, diabetes, hyperlipidemia, IPAQ.
